# Calcification caséeuse de l’anneau mitral: à propos d’un cas

**DOI:** 10.11604/pamj.2026.53.120.47736

**Published:** 2026-03-10

**Authors:** Wiem Feki, Amin Bahloul, Slim Bouaziz, Imen Haddar, Emna Daoud, Leila Abid, Salma Charfeddine

**Affiliations:** 1 Département de Radiologie, Centre Hospitalo-Universitaire Hédi Chaker, Sfax, Tunisie,; 2 Département de Cardiologie, Centre Hospitalo-Universitaire Hédi Chaker, Sfax, Tunisie,; 3 Département de Radiologie, Centre Hospitalo-Universitaire Habib Bourguiba, Sfax, Tunisie

**Keywords:** Calcification caséeuse, anneau mitral, imagerie multimodale, cas clinique, Caseous calcification, mitral annulus, multimodal imaging, case report

## Abstract

La calcification caséeuse de l'anneau mitral est une entité rare et souvent méconnue des cliniciens, y compris des radiologues. Son diagnostic peut s'avérer difficile en échocardiographie, avec un risque de confusion avec des tumeurs ou pseudomasses cardiaques. Dans ce contexte, nous rapportons le cas d'une patiente de 40 ans, aux antécédents médicaux notables d'hypertension artérielle et d'insuffisance rénale chronique, hospitalisée pour une pneumopathie infectieuse compliquée de douleurs thoraciques droites. Une échocardiographie réalisée dans le cadre du bilan a révélé un rétrécissement aortique serré, une calcification de l'anneau mitral, une insuffisance mitrale modérée et une insuffisance tricuspide sévère. En vue d'une évaluation préopératoire, un coroscanner a été effectué, mettant en évidence une formation hypodense au niveau du trigone aortomitral, compatible avec une calcification caséeuse de l'anneau mitral. Ce cas illustre la complexité diagnostique en présence d'anomalies valvulaires multiples associées à des facteurs de risque cardiovasculaires et métaboliques. Il souligne également l'intérêt crucial d'une imagerie multimodale pour affiner le diagnostic différentiel et optimiser la stratégie thérapeutique. Ce cas contribue ainsi à enrichir la littérature médicale sur la prise en charge des calcifications annulaire et caséeuse.

## Abstract (en)

Caseous calcification of the mitral annulus is a rare entity, often unrecognised by clinicians and radiologists alike. It can be difficult to diagnose on echocardiography, with the risk of confusion with cardiac tumours or pseudomasses. In this context, we report the case of a 40-year-old female patient with a notable medical history of arterial hypertension and chronic renal failure, admitted to the hospital with infectious pneumonitis complicated by right-sided chest pain. An echocardiogram performed as part of the work-up revealed severe aortic narrowing, calcification of the mitral annulus, moderate mitral insufficiency and severe tricuspid insufficiency. For pre-operative assessment, a coroscan was performed, demonstrating a hypodense formation in the aortomitral trigone, consistent with caseous calcification of the mitral annulus. This case illustrates the diagnostic complexity of multiple valve anomalies associated with cardiovascular and metabolic risk factors. It also highlights the crucial importance of multimodal imaging in refining the differential diagnosis and optimising the therapeutic strategy. This case thus contributes to the medical literature on the management of annular and caseous calcifications.

## Introduction

La calcification caséeuse de l'anneau mitral (CCAM) est une entité rare et méconnue, représentant une forme particulière de calcification annulaire mitrale (CAM), souvent découverte fortuitement à l'imagerie. Contrairement à la CAM classique, la CCAM se distingue par son aspect pseudotumoral, pouvant prêter à confusion avec d'autres masses intracardiaques, ce qui rend son identification cruciale pour éviter des diagnostics erronés. Son association fréquente avec des comorbidités cardiovasculaires et métaboliques, notamment l'insuffisance rénale chronique, souligne l'importance de la reconnaître, d'autant plus que son diagnostic repose essentiellement sur une imagerie cardiaque experte. Le cas présenté ici illustre cette pathologie et met en lumière l'intérêt d'un diagnostic précis et documenté pour une meilleure orientation thérapeutique.

## Patient et observation

**Information relative à la patiente:** il s'agit d'une patiente âgée de 44 ans aux antécédents de tabagisme actif à 30 paquets par année, fibrillation auriculaire paroxystique, hypertension artérielle, insuffisance rénale chronique au stade d'hémodialyse.

**Résultats cliniques:** elle a été hospitalisée au service de pneumologie pour une pneumopathie infectieuse.

**Figure 1:**
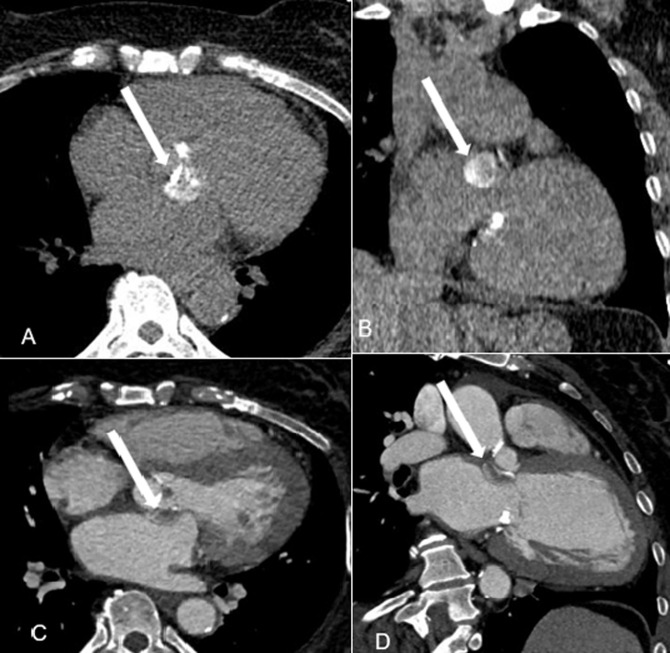
A, B, C, D) aspect scanographique de la calcification caséeuse de la valve mitrale

**Chronologie et démarche diagnostique:** au cours de son hospitalisation, la patiente a présenté des douleurs thoraciques ayant motivé la réalisation d'une échographie transthoracique qui a montré un rétrécissement aortique serré (SAo = 0,6 cm^2^), un anneau mitral calcifié, une insuffisance mitrale modérée et une insuffisance tricuspide sévère. Une coronarographie a été programmée dans le cadre du bilan préopératoire d'un remplacement valvulaire aortique et d'une plastie tricuspide, toutefois, le guide n'a pas pu franchir l'artère radiale probablement par la présence d'une infiltration athéromateuse sévère. Un coroscanner a été alors réalisé et a montré (Figure 1): i) Une calcification coronaire avec un score de calcique à 177 UH (unité Hounsfield); ii) Une calcification sévère mitrale et aortique avec des scores calciques mesurant respectivement 3992 et 3629; iii) Une formation hypodense de l'espace intervalvulaire précisément au niveau du trigone aortomitral, présentant une calcification fine périphérique, sans rehaussement après injection de produit de contraste, mesurant 25 x 10 mm dans le plan axial et 23 mm en coronal. Cette masse évoque une calcification caséeuse de l'anneau mitral.

**Interventions thérapeutiques:** aucune intervention n'a été faite vu qu'il s'agit d'un terrain à risque et le refus total de la patiente pour toute intervention chirurgicale.

**Suivi:** un contrôle annuel par imagerie et notamment un scanner thoracique sans injection de produit de contraste *low dose* est programmé.

**Consentement du patient:** le consentement éclairé pour la publication des données cliniques a été obtenu de la patiente.

## Discussion

La calcification de l'appareil mitral peut toucher l'anneau mitral (calcification annulaire mitrale ou « CAM »), les feuillets ou l'appareil sous-valvulaire (cordages et muscles papillaires). Parmi ces formes, la CAM est la plus fréquente, avec une prévalence d'environ 6% dans la population générale [1]. Elle correspond à un processus dégénératif chronique de la base fibreuse de la valve mitrale, associé au vieillissement, mais également à des mécanismes athéroscléreux actifs. Des facteurs tels qu'un stress valvulaire accru (sténose aortique, hypertension), ainsi que des troubles du métabolisme phosphocalcique, notamment en cas d'insuffisance rénale chronique, peuvent en favoriser le développement.

La CCAM, encore connue sous le nom de *Caseous Mitral Annulus Calcification* des anglo-saxons (CMAC) est une forme peu connue et rarement décrite de la CAM. La terminologie de la CCAM est particulière, car le terme « caséeuse » fait généralement référence à un type de nécrose souvent observé dans la tuberculose [2]. La prévalence exacte de la CCAM reste inconnue, souvent sous-diagnostiquée en raison d'un manque de familiarité des praticiens avec cette pathologie et de la sensibilité limitée de l'échocardiographie. Les études montrent une différence de prévalence entre les données échocardiographiques et celles issues des autopsies. Toutefois, avec l'amélioration et la généralisation des techniques d'imagerie cardiaque, le nombre de cas diagnostiqués est en augmentation [3]. La CCAM se présente sous la forme d'une masse arrondie, parfois semi-lunaire, de grande taille, hyperéchogène, à consistance molle, avec des zones centrales hypoéchogènes. Elle est visible en échocardiographie transthoracique et, surtout, transœsophagienne, ressemblant à une masse péri-annulaire. Elle se situe au niveau de la portion postérieure de l'anneau mitral, contrairement à la CAM classique qui touche généralement la base moyenne du feuillet postérieur, mais peut aussi affecter d'autres segments de l'anneau mitral [4].

Dans la CAM, la calcification débute habituellement entre le sillon atrioventriculaire postérieur et la base du feuillet mitral postérieur. Chez les sujets âgés, elle peut s'étendre à tout l'anneau. La CCAM représente une évolution rare de l'anneau mitral calcifié, liée à une transformation caséeuse du matériel interne [4]. Le mécanisme précis impliqué dans la liquéfaction et la caséification dans la CCAM n'est pas encore bien compris. Étant donné que la prévalence de la CCAM est plus élevée chez les patients atteints d'insuffisance rénale terminale, en particulier chez ceux sous dialyse, un trouble du métabolisme du calcium et du phosphate est impliqué dans sa pathogenèse [2].

La découverte fortuite d'une masse intracardiaque lors d'un examen d'imagerie cardiaque constitue le mode de révélation le plus fréquent de la CCAM comme pour notre patiente. Toutefois, les symptômes les plus fréquemment rapportés par les patients sont les palpitations et la dyspnée (secondaires à une congestion veineuse pulmonaire), plus rarement, certains patients peuvent présenter une syncope (due à des blocs auriculoventriculaires) [5]. Des complications emboliques systémiques peuvent survenir au cours de la CCAM, telles que des accidents vasculaires cérébraux, une occlusion de l'artère rétinienne ou un syndrome coronarien aigu. Les mécanismes supposés de ces embolies comprennent : l'embolie de petites particules calcifiées, l'ulcération de la surface avec formation d'un thrombus, suivie d'une embolie, ou encore la fistulisation du matériel nécrotique caséeux dans la lumière de l'oreillette ou du ventricule gauche [6].

À l'imagerie, l'échocardiographie constitue le premier examen d'imagerie pour évaluer la calcification caséeuse de la valve mitrale permettant d'identifier ses caractéristiques typiques avec la localisation habituelle dans la région annulaire postéro-latérale de la valve mitrale, des bords lisses et un aspect globalement hyperéchogène avec des zones centrales hypoéchogènes [5]. Pour notre patiente, la CMAC n'a pas été visualisée lors de l'échographie. Dans les cas incertains, l'utilisation de l'imagerie par résonance magnétique (IRM) ou mieux de la tomodensitométrie (TDM) s'avère utile [6]. La CMAC se caractérise par un noyau hypointense sur les séquences T1 et T2, ce qui fait la différence avec les autres diagnostics, tels que le myxome et le lipome qui présentent une hyperintensité centrale sur les séquences T2 en raison de leur stroma mucineux ou adipeux. De même, les abcès myocardiques présentent une hyperintensité centrale entourée d'un anneau hypo-intense, bien que ces caractéristiques varient selon le stade et le type d'abcès [7]. Par ailleurs, la CMAC se présente comme une masse avasculaire, ne présentant aucun rehaussement après l'injection de gadolinium, que ce soit sur les séquences de perfusion ou en rehaussement tardif [8].

Bien que l'IRM soit une technique de référence pour la caractérisation tissulaire, sa sensibilité aux artefacts - notamment ceux induits par la respiration, les mouvements cardiaques ou les arythmies - peut en limiter la précision diagnostique. De plus, sa disponibilité souvent restreinte et la complexité de sa planification, comparée à la TDM, constituent des obstacles à son utilisation systématique en pratique clinique. La distinction entre certaines masses intracardiaques peut également rester difficile: les thrombi peuvent présenter des caractéristiques similaires en IRM, tandis que certains myxomes montrent des signes partiels de vascularisation ou de nécrose. Ainsi, en cas de doute diagnostique, des examens d'imagerie complémentaires peuvent s'avérer nécessaires [9].

La TDM cardiaque, quant à elle, offre une excellente visualisation de la structure typique de la CCAM, mettant en évidence un centre hypodense avasculaire, entouré d'une capsule fibreuse et d'un rebord calcifié de manière irrégulière. Toutefois, l'identification pathognomonique de cette lésion nécessite des acquisitions avant et après injection de produit de contraste, ce qui expose le patient à une dose significative de rayonnement [10]. Ainsi, en raison des contraintes liées à l'IRM (durée d'acquisition prolongée) et des risques d'irradiation associés à la TDM, ces examens doivent être réservés aux situations où l'échographie ne permet pas d'établir un diagnostic formel de CCAM [6].

## Conclusion

La CCAM est une forme rare et souvent méconnue de la calcification annulaire mitrale, généralement découverte fortuitement à l'imagerie. Elle est fréquemment associée à des facteurs de risque cardiovasculaires et métaboliques, notamment l'insuffisance rénale terminale. Son diagnostic repose principalement sur l'échocardiographie, complétée si besoin par l'IRM ou au mieux par la TDM pour mieux caractériser la masse. Une bonne connaissance de ses caractéristiques permet d'éviter les confusions avec d'autres masses intracardiaques et d'adapter la prise en charge.

## Remerciements

Nous remercions le chef de service de radiologie du CHU Hédi Chaker de Sfax, Dr Zeineb Mnif, qui encourage toute l'équipe au travail et à la publication des articles scientifiques.
